# CGH and SNP array using DNA extracted from fixed cytogenetic preparations and long-term refrigerated bone marrow specimens

**DOI:** 10.1186/1755-8166-5-10

**Published:** 2012-02-02

**Authors:** Ruth N MacKinnon, Carly Selan, Adrian Zordan, Meaghan Wall, Harshal Nandurkar, Lynda J Campbell

**Affiliations:** 1 Victorian Cancer Cytogenetics Service, St Vincent's Hospital (Melbourne), Fitzroy, Vic, Australia; 2 Department of Medicine (St Vincent's Hospital, Melbourne), University of Melbourne, Australia; 3 Immunology Research Centre, St. Vincent's Hospital, Melbourne, Australia; 4 Department of Haematology, St Vincent's Hospital, Melbourne, Australia

**Keywords:** SNP array, array CGH, bone marrow, archived specimens, old archived specimens, DNA extraction, DNA analysis, U937

## Abstract

**Background:**

The analysis of nucleic acids is limited by the availability of archival specimens and the quality and amount of the extracted material. Archived cytogenetic preparations are stored in many laboratories and are a potential source of total genomic DNA for array karyotyping and other applications. Array CGH using DNA from fixed cytogenetic preparations has been described, but it is not known whether it can be used for SNP arrays. Diagnostic bone marrow specimens taken during the assessment of hematological malignancies are also a potential source of DNA, but it is generally assumed that DNA must be extracted, or the specimen frozen, within a day or two of collection, to obtain DNA suitable for further analysis. We have assessed DNA extracted from these materials for both SNP array and array CGH.

**Results:**

We show that both SNP array and array CGH can be performed on genomic DNA extracted from cytogenetic specimens stored in Carnoy's fixative, and from bone marrow which has been stored unfrozen, at 4°C, for at least 36 days. We describe a procedure for extracting a usable concentration of total genomic DNA from cytogenetic suspensions of low cellularity.

**Conclusions:**

The ability to use these archival specimens for DNA-based analysis increases the potential for retrospective genetic analysis of clinical specimens. Fixed cytogenetic preparations and long-term refrigerated bone marrow both provide DNA suitable for array karyotyping, and may be suitable for a wider range of analytical procedures.

## Background

Array comparative genomic hybridization (array CGH) [1] and single nucleotide polymorphism (SNP) array [2] are array-based karyotyping techniques which can help determine the genome abnormalities causing genetic disorders and the acquired genome copy number changes in cancer cells. They provide higher resolution than traditional karyotyping. However, the use of these and other DNA-based approaches for analysis is sometimes limited by the availability of suitable tissue samples, particularly for retrospective cancer genome analysis.

Fixed cytogenetic specimens are often stored after analysis, and are a potential source of total genomic DNA for array karyotyping and other DNA analysis techniques. Bone marrow specimens are commonly used to determine karyotype abnormalities in hematological malignancies. If unprocessed bone marrow is stored at 4°C during this time, it may be up to a month old before a karyotype is known and the decision to carry out array karyotyping is made. Anecdotally, it has been assumed that DNA extracted from these types of specimen is too degraded for analysis. Here we show that, on the contrary, an array karyotyping result can be obtained from these specimens.

The process of fixing and storing cells in 3:1 methanol/acetic acid introduces the possibility of acid nicking and degradation of the DNA. Two groups have reported array CGH using total genomic DNA extracted from cytogenetic preparations [3,4]. To our knowledge this approach is not widely known.

Here we present a modified protocol for total genomic DNA extraction from fixed cytogenetic preparations, which addresses the need to obtain an optimum yield and concentration from a finite amount of starting material. We show that this DNA produces array CGH results of high quality, and we describe for the first time the use of DNA extracted from this source for SNP array. We also show that bone marrow that has been refrigerated (not frozen) for over a month yields DNA that, although partially degraded, produces reliable SNP array and array CGH results..

## Results

**Figure 1:**
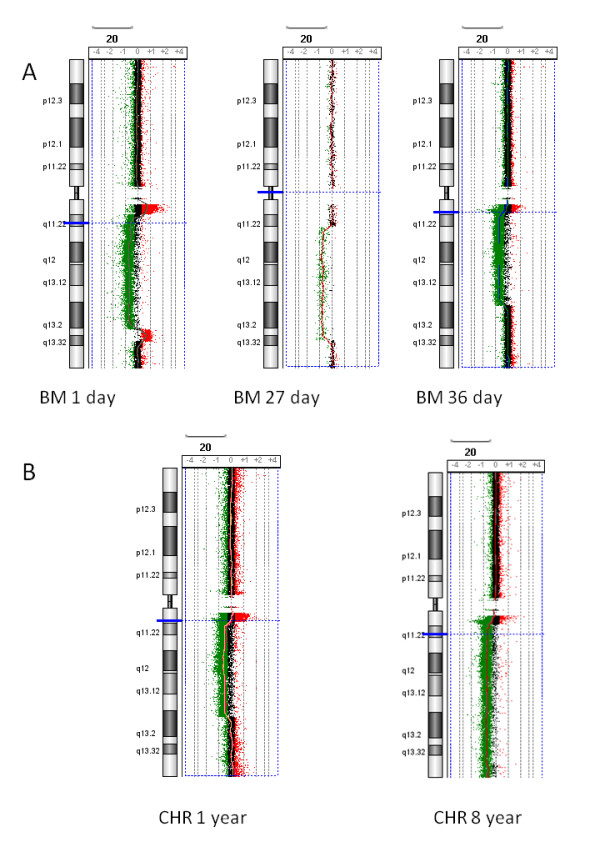
**Examples of array CGH**. A. Images produced using DNA extracted from bone marrow (BM) refrigerated for 1 day (diagnosis specimen of SVH05 [8]), 27 days (AML specimen from [7]) and 36 days. B. Images produced from fixed cytogenetic preparations (CHR) stored at -80°C for one year and 8 years. All of these 20q deletions were validated with the 20q12 Vysis probe, LSI D20S108 (20q12) SpectrumOrange. A 2 Mb moving average line is shown for each experiment. Each image represents duplicate experiments except for the 27 day old bone marrow, which represents one experiment. The catalog 60K array used for the BM 27 day result which has a median probe spacing of 41 kb and the other results are from a Agilent 44K custom array with probes 200 bp (20q11.21- > 20q11.22), 5 kb (20q11.22- > 20q12) and 9 kb (20p and 20q13- > 20qter) apart.

**Figure 2:**
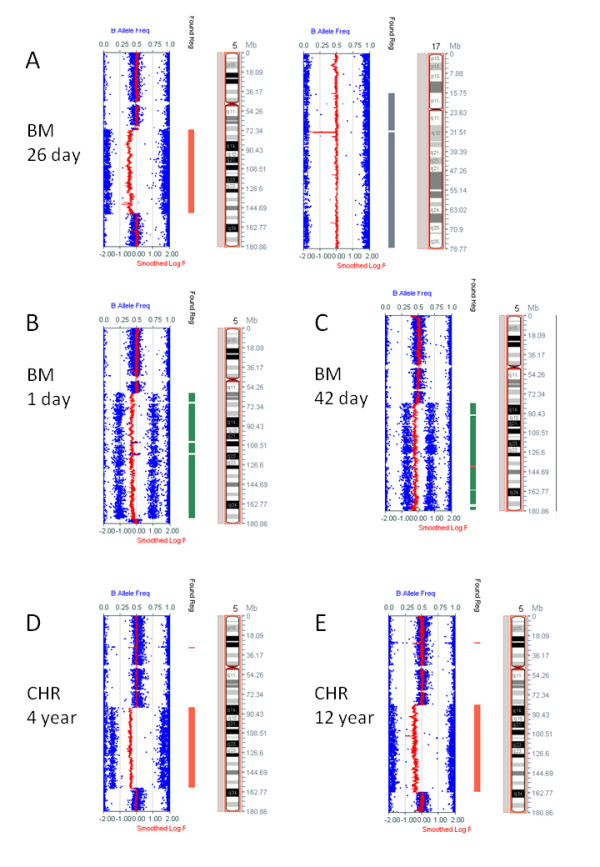
**Examples of SNP array images**. A. From bone marrow (BM) refrigerated for 26 days before DNA extraction (Case SVH01 of [8]) showing deletion of 5q (left) and copy number neutral LOH of chromosome 17 (right). B-C. From bone marrow refrigerated for (B) one day and (C) 42 days before DNA extraction. D-E From DNA extracted from (D) 4 year old and (E) 12 year old fixed cytogenetic preparations (CHR). Deletion of 5q in each example was validated by FISH. In the case shown in A there were two copies of a dic(17;20) and the un-rearranged chromosome 17 had been lost [18], affirming copy number neutral LOH of chromosome 17. The CytoSNP 12 microarray has a 6.2 kb median probe spacing.

**Figure 3:**
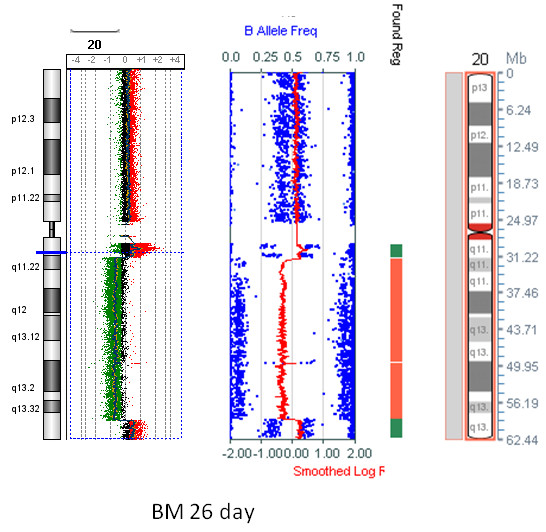
**A comparison of array CGH and SNP array results for chromosome 20 from 26 day old bone marrow**. Bone marrow was refrigerated for 26 days before DNA extraction. Deletion, gain or amplification of different regions of 20q, and low level gain of 20p have been extensively validated by single locus FISH, G-banding, M-BAND and M-FISH (Case SVH01, [8]). Duplicate experiments were performed, and two 0.2 Mb moving average lines are shown for the array CGH specimen to show the peaks of localized amplification. The probes in the custom Agilent array are spaced between 200 bp and 9 kb apart (see Figure 1) and the Illumina CytoSNP 12 array probes have a median spacing of 6.1 kb.

**Figure 4:**
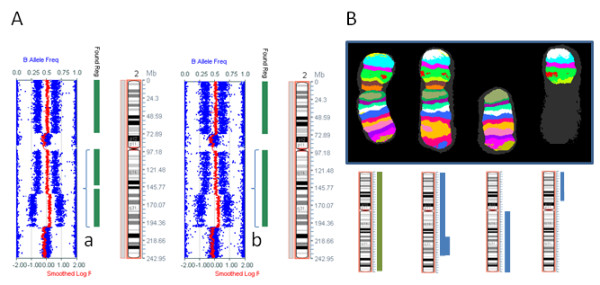
**A comparison of SNP array results for chromosome 2 using DNA from fresh and fixed cells**. A. A comparison of SNP array results from fresh (left) and fixed (right) U937 cell line. Gain of a section of the long arm (brackets a, b) is denoted by a single vertical green bar for the fixed specimen (Found Reg = Found Region) (b) whereas in the fresh specimen (a) this is divided into two separate sections. This is representative of the minor boundary differences determined by the Karyostudio software, between the two experiments. B. The M-BAND pattern for chromosome 2. The idiograms below the banded chromosomes show the section of chromosome 2 present in each chromosome. The green bar represents the homolog from one parent and the blue bars represent the homolog from the other parent (inferred from B allele frequencies, see Methods). The Illumina CytoSNP 12 array probes have a median spacing of 6.1 kb.

**Figure 5:**
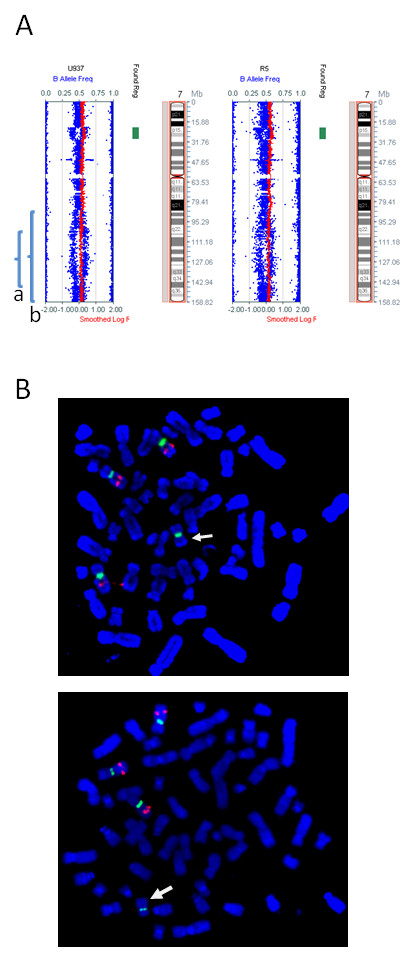
**A comparison of SNP array results for chromosome 7 using DNA from fresh and fixed cells**. A. A comparison of SNP array results from fresh (left) and fixed (right) U937 cell line. The brackets on the left show (a) a 38 Mb deletion and (b) a 70 Mb deletion of 7q which encompasses (a). B. Cells with the smaller (top) and larger (bottom) 7q deletion validated by FISH with the Vysis LSI D7S486 SpectrumOrange (7q31, red) and CEP7 SpectrumGreen (centromere, green) probes in metaphase spreads. The deleted chromosome 7 is arrowed. The Illumina CytoSNP 12 array probes have a median spacing of 6.1 kb.

To date we have used DNA extracted from cytogenetic preparations to perform five array CGH experiments and four SNP array experiments. We have also used DNA extracted from bone marrow specimens refrigerated for nine or more days, for nine array CGH experiments (five of these bone marrow specimens were stored at 4°C for 25-36 days before DNA extraction) and 25 SNP array experiments (eight were stored at 4°C for 25-42 days before DNA extraction). Representative results are shown in Figures 1, 2, 3, 4 &5.

Selected copy number aberrations detected by SNP array and array CGH were validated by FISH (See Figures 1, 2, 3, 4 &5). Chromosome 20 deletions were confirmed with a probe for the 20q12 deletion marker *D20S108 *(Vysis LSI D20S108 (20q12) SpectrumOrange). Chromosome 5q deletions were confirmed by multicolour FISH (M-FISH) and/or multicolour banding (M-BAND) and a probe detecting 5q31 deletions (Vysis LSI EGR1 (5q31) SpectrumOrange/D5S721, D5S23 SpectrumGreen, Abbott Molecular).

### DNA size and yield

**Figure 6:**
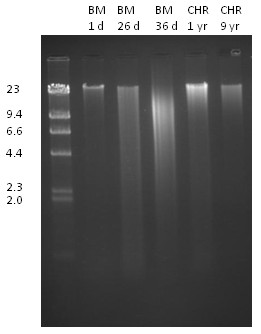
**An agarose gel showing sizes of DNA extracted from bone marrow and fixed cytogenetic preparations**. A. A 0.7% agarose gel showing representative total genomic DNA prepared from bone marrow specimens and chromosome suspensions. BM, bone marrow refrigerated for one day (160 ng); 26 days (235 ng); and 36 days (100 ng); CHR, fixed cytogenetic preparations stored at -80°C for one year (80 ng); and nine years (40 ng). The molecular weight marker (left) is λ/HindIII and the sizes of its bands are indicated on the left in kb.

The agarose gel in Figure 6 shows representative DNA samples from the two types of specimens of various ages. The size of the DNA extracted from bone marrow decreased with the length of time the unprocessed bone marrow had been stored at 4°C, and averaged less than 20kb after 36 days (Figure 6). Fixation of the cells in 3:1 methanol/glacial acetic acid led to some degradation of the DNA compared to DNA prepared from one day old bone marrow. Long-term storage of the chromosome preparations at -80°C did not produce further degradation (Figure 6).

DNA yield from chromosome suspensions was in the range of 2-4 μg per 10^6 ^nuclei. Optical densities (OD_260/280_) were consistently at or above 1.8, which is the recommended purity for both the Agilent and Illumina microarray platforms. Qiagen recommends two 200 μL elutions for maximum yield from the DNeasy Cell and Tissue Kit. For low cellularity bone marrow and chromosome preparations, 200 μL elutions yielded DNA that was too dilute for the CGH or array protocols (e.g. < 10 ng/μL). We established that DNA could be extracted from a minimum of 10^6 ^fixed cells. By estimating the cellularity of chromosome preparations and using a lower first elution volume (40 μL) we were able to obtain DNA at a suitable concentration. From 1 × 10^6 ^fixed cells from a valuable specimen we obtained 69 ng/μL with a total yield greater than 4 μg.

### Quality metrics

The DNA Workbench software for Agilent array CGH has inbuilt Quality Control (QC) metrics. The DLRSpread (Derivative Log Ratio Spread) is a measure of hybridization specificity. The ranges of values used to define "Excellent", "Good" and "Poor" by DNA Workbench are listed in Table 1.

**Table 1:** Quality Control Metrics from DNA Workbench

Specimen	**Age of specimen**^**1**^	Test Fluorochrome	Pass/fail	**DLRSpread**^**2**^	Signal to Noise ^3 ^Green	Signal to Noise ^3 ^Red
bone marrow	1 day	CY3	Pass	0.206803	65.848474	63.860383
		CY5	Pass	**0.199919**	62.305342	52.162212
bone marrow	9 days	CY3	Pass	**0.14503**	98.418512	76.505274
		CY5	Pass	**0.142963**	87.635554	88.5432
bone marrow	15 days	CY3	Pass	0.209813	44.387109	34.835231
		CY5	Pass	0.205444	53.479156	41.008914
bone marrow	26 days	CY3	Pass	0.283387	38.740122	43.751865
		CY5	Pass	0.257136	39.285321	42.250693
bone marrow	27 days	CY3	Pass	**0.158672**	85.404253	76.821745
bone marrow	36 days	CY3	Pass	**0.181903**	71.365826	75.92553
		CY5	Pass	**0.192083**	59.676813	56.174289
						
cytogenetic specimen	52 days	CY3	Pass	**0.174283**	**123.04883**	**104.706454**
cytogenetic specimen	1 year	CY3	Pass	0.333462	40.65296	49.663864
		CY5	Pass	**0.193984**	56.378169	43.05297
cytogenetic specimen	8 years	CY3	Pass	0.218933	47.366763	47.266465
		CY5	Pass	**0.198984**	57.315046	56.775467

DNA Workbench quality classifications are given below the table. Quality measurements classified as **"excellent" **are in bold print and the single "poor" measurement is underlined; all other measurements were classified "good". Pairs of duplicate experiments are grouped together.

^1 ^length of time at 4°C for bone marrow or at -80°C for chromosome suspension in Carnoy's fixative.

^2 ^Excellent: < 0.2; Good: 0.2-0.3; Poor: > 0.3

^3 ^Excellent: > 100; Good: 30-100; Poor: < 30

DLRSpread values for array CGH from both fixed cytogenetic preparations (DLRSpread = 0.17-0.33) and longer-term refrigerated bone marrow specimens (DLRSpread = 0.16-0.28 for > 14 days at 4°C) compared favourably with those obtained using DNA extracted from fresher bone marrow (DLRSpread 0.14-0.20 for < 10 days at 4°C) (Table 1). The QC metrics for these specimens typically fell within the ranges "Good" to "Excellent", and included "Excellent" DLRSpread values for DNA extracted from bone marrow refrigerated for more than 26 days (Table 1). DNA extracted from bone marrow that had been refrigerated for 36 days also produced among the best DLRSpread values. The single specimen giving a "Poor" DLRSpread value obtained in this series of experiments, obtained from a one year old fixed cytogenetic preparation, also gave an "Excellent" DLRSpread value in a duplicate experiment; the overall result for this specimen was a "Pass". Therefore, the array CGH protocol can tolerate the level of DNA degradation that occurred in our specimens. Our results show that these specimens can be reliably used for array CGH.

There is no equivalent measure of SNP array signal spread or efficiency available in the Illumina Karyostudio software. However, SNP array images produced from both cytogenetic preparations and long-term refrigerated bone marrow showed low background and scatter (Figure 2).

### Comparison of SNP array results from fresh and fixed cells

DNA was extracted from both live and fixed myeloid cell line U937 cells prepared from the same culture flask, for comparison of SNP array results. U937 contains copy number aberrations of all chromosomes except chromosome 9 from a basically triploid karyotype ([5]; R. MacKinnon: A detailed molecular karyotype of the myeloid cell line U937 using combined FISH, M-FISH, M-BAND and SNP array, manuscript in preparation), allowing a comparison of the copy number aberrations and loss of heterozygosity (LOH) detected at many sites across the genome from fresh and fixed cells. The SNP array results displayed in Illumina Karyostudio matched the M-FISH pattern for this cell line (RM, unpublished results) and the copy number aberrations on chromosome 2 in U937 were confirmed by M-BAND (Figure 4).

Comparison of fresh and fixed specimen images for the same chromosomes revealed an increased spread of the B allele frequency (BAF) scatter plot from the fixed cells, which did not affect the result. It should be noted that the U937 cells were processed directly from live cells, whereas all bone marrow specimens analysed were stored at 4°C for at least one day before DNA extraction.

The same copy number aberrations were identified in fresh and fixed U937 tissue. There were minor variations in the boundaries called by the Karyostudio algorithm, an example of which is shown in Figure 4, in which a region of 2q gain was called as two distinct regions in the fresh specimen and a single region in the fixed specimen.

Some copy number aberrations could only be identified by visual examination of the B allele frequencies (an example shown in Figure 5). Mosaicism for a 7q deletion in the U937 specimen allowed us to assess the level of sensitivity of the SNP array for both fresh and fixed specimen. Two sub-clones in our U937 culture had overlapping deletions of 7q from one of four chromosomes 7. The overlapping deleted region (region a, Figure 5A) was identified in both the fresh and fixed U937 specimens, with BAF values of about 0.4 and 0.6 (equivalent to 66.7% cells with a 7q deletion on a tetrasomic background of AABB). The deletion was confirmed by FISH in metaphase nuclei and 207/300 (69%) interphase nuclei (Figure 5B). A deletion in 1/3 diploid cells would give the same BAF values. The larger deletion (region b, Figure 5A) was observed in 31% of metaphases (16/51), a frequency which would produce BAF values of 0.54 and 0.46 (equivalent to a deletion in 15% of diploid cells). This change in BAF values was apparent but not unequivocal in either specimen.

## Discussion

We have assessed the use of two types of archived specimens for both array CGH and SNP array. Cytogenetic preparations from diagnostic analysis are often archived in laboratory freezers and should be seen as a potential source of archival material for research into the genetics of malignancy and inherited disease. In our laboratory, fixed cytogenetic preparations are often the only stored patient specimen available. Bone marrow specimens received for karyotyping are also a potential source of DNA, but it has been assumed that the DNA is not suitable unless processed or frozen immediately. We have shown that these specimens can be successfully used for array karyotyping.

In 1986 Barker *et al. *[6] reported that DNA suitable for Southern analysis can be extracted from fixed cytogenetic preparations, using a phenol/chloroform protocol, and they suggested that this DNA might be suitable for other analytical purposes. Our array CGH results confirm the use of DNA extracted from fixed cytogenetic preparations for array CGH [3,4,7], and here we show that the quality metrics of the results compare favourably with results from one day old bone marrow. We have shown for the first time that DNA from fixed cytogenetic preparations can also be used for SNP array. DNA prepared from fixed chromosomes was slightly degraded but was not degraded further with longer storage at -80°C (Figure 6).

We describe a modified DNA extraction method for use with cytogenetic suspensions. By processing a known cell number and performing an initial lower volume elution, the amount of specimen used can be kept to a minimum. This is important for limited volume specimens or specimens with low cellularity. In low cellularity specimens this produced an eluant that could be used without further concentration and loss of DNA. Interestingly we have managed to extract a small amount of highly degraded RNA from fixed cytogenetic suspensions which have been used successfully for Real-Time PCR [8].

Validation of array CGH and SNP array results showed that they were reliable. Direct comparison of the same specimen processed fresh or fixed showed only a small increase in the spread of B allele frequencies in results derived from a fixed specimen when compared with the same cell line processed fresh. A deletion producing a shift in BAF values from 0.5 to 0.4 and 0.6 was clearly visible in both specimens (equivalent to a deletion in 1/3 of diploid cells).

We also achieved reliable SNP array and array CGH results using unprocessed bone marrow specimens refrigerated for 36 days or more before freezing. Agarose gel analysis showed that the DNA in bone marrow stored at 4°C degraded over time (see Figure 6). However, specimens stored for up to 36 or 42 days at 4°C were still suitable for both array CGH and SNP array, respectively.

As it may occasionally take weeks to determine a karyotype, considerable time and resources can be saved if only the specimens of interest are frozen or processed after a karyotype is known. This approach is particularly suitable for bone marrow, which is difficult to re-collect. Also, therapy may have been administered or the genotype of malignant cells may have changed by the time a subsequent collection is contemplated.

Cytogenetic slides are another potential source of DNA for microarray studies. A method has been described for extracting DNA from chromosomes scraped from microscope slides for PCR [9]. However, around 10^6 ^nuclei are needed for the extraction protocol we describe, and so a single cytogenetic slide would not produce enough DNA for array karyotyping without amplification. Thus, while DNA could potentially be obtained from slides, use of unspread specimens is simpler, and is less likely to require amplification.

Other methods of DNA analysis may also be possible using DNA extracted from these specimens. DNA extracted from formalin-fixed paraffin embedded tissues (FFPE) can be used for array CGH and SNP array analysis, although sensitivity is much poorer than for fresh tissue [10-12]. Whole genome amplification makes it possible to use a small starting amount of DNA [10,12]. Improved protocols make sequencing of degraded DNA extracted from FFPE specimens possible [13,14], suggesting that DNA from cytogenetic preparations will be even more suitable for this and other methods of DNA analysis. Massively parallel sequencing can be carried out on as few as six microdissected and amplified chromosome segments [15].

## Conclusions

We have shown that cytogenetic preparations in long-term storage, and bone marrow specimens which have been refrigerated unprocessed for at least 36 days, can be used as a source of genomic DNA for both SNP array and array CGH. We have also described a modified DNA extraction protocol for use with cytogenetic preparations of low cellularity. These methods will prove particularly useful for cancer genome analysis. They will allow chromosome abnormalities at a certain point in disease evolution to be studied retrospectively, and make retrospective analysis possible for patients for whom there are no alternative archival specimens.

## Methods

### Specimens

The U937 cell line is a myeloid leukemia cell line [5,16]. All other specimens were from patients with myeloid malignancies (myelodysplastic syndromes or acute myeloid leukemia).

### DNA extraction

All DNA extractions were performed with a DNeasy Cell and Tissue Kit (Qiagen, Germantown, MD), using the blood protocol or a modification thereof.

Bone marrow from patients with myeloid malignancies was collected in tubes containing 100 IU (1,000 IU/mL) sodium heparin and sent to the Victorian Cancer Cytogenetics Service (VCCS) for cytogenetic analysis. Residual specimen that was not used for the preparation of fixed cell suspensions was stored at 4°C. After determination of the karyotype, residual bone marrow specimens of potential use for array karyotyping studies were transferred to cryotubes and stored at -80°C. Total genomic DNA was prepared subsequently from 100 μL thawed whole bone marrow specimen.

Total genomic DNA was also prepared from fixed cytogenetic suspensions which had been prepared from cultured bone marrow cells using standard techniques (hypotonic treatment followed by lysis, the addition of 3:1 methanol:acetic acid [17]) and stored at -80°C for up to twelve years. Cell concentration was estimated by thorough resuspension and spreading of three 1 μL aliquots from separate known dilutions on a clean glass slide. Interphase and metaphase nuclei were counted and averaged. Cytogenetic suspensions containing at least 10^6 ^nuclei were rinsed three times with PBS, resuspended in 200 μL PBS and processed using the DNeasy Cell and Tissue Kit without RNase treatment. However, when specimens were of low cellularity, the recommended 200 μL elution produced specimens which were too dilute for accurate quantitation and direct use in the array protocols. Therefore, instead of the recommended 200 μL elution, two or more elutions of 40-100 μL, for at least 10 minutes each, were carried out with the AE eluant provided in the kit. The lower volume first elution was chosen if the amount of specimen was limited (less than 10^7 ^nuclei), to ensure a usable final concentration. Further elutions were used to recover more of the residual DNA from the column.

The U937 cell line [5,16] (obtained from the laboratory of Hamish Scott, Walter and Eliza Hall Institute, Melbourne), was cultured in RPMI containing 10% FCS, glutamine, penicillin and streptomycin at 37°C in air containing 5% CO_2_. Fresh cultured U937 cells were split into two equal volumes and immediately processed. DNA was extracted by two different methods, to allow a comparison between DNA extracted from fresh and fixed tissue: (1) DNA was extracted directly from cultured cells using the Qiagen DNeasy kit blood protocol, or (2) metaphases were harvested according to standard cytogenetics protocols, stored at -80°C for 70 days, and DNA extracted with the DNeasy Cell and Tissue kit using the protocol described above.

DNA was run on 0.7% agarose to check for integrity and quantitated with a Nanodrop spectrophotometer (Thermo Scientific, Wilmington, DE).

### Array karyotyping - SNP array

Illumina CytoSNP 12 arrays (Illumina, San Diego, CA) were processed according to the manufacturer's instructions using 200 ng of each DNA sample in a 4 μL volume. Data were analysed using Karyostudio (version 1.2, Illumina).

B allele frequencies were used to determine copy numbers of chromosome 2 regions in the abnormal chromosomes represented in Figure 4. The SNP array pattern (log R ratio and B allele frequency) is consistent with (from p arm to q arm) 3-2-3-4-2 copies of each region. The B allele frequencies show that at each region there is one copy of one homolog and 1-3 copies of the other homolog, which is consistent with all rearrangements occurring in one of the homologs (Figure 4).

### Array karyotyping - array CGH

Array CGH was carried out using the Agilent platform according to the manufacturer's instructions. Custom 44K and 105K arrays with a high probe density on chromosome 20 were used for all but the 27 day old bone marrow specimen (see Table 1) which was a catalog 60K array (design 021924). Test and control (Promega pooled Human Genomic DNA of the opposite sex) DNA were labelled with Cyanine 3-dUTP and Cyanine 5-dUTP using an Agilent Genomic DNA Labeling Kit PLUS (Agilent, Santa Clara, CA), denatured and co-hybridized before hybridizing to a custom 44K array. Data were analysed using the Genomic Workbench software (version 5.0.14, Agilent) and regions of significant gain or loss were determined using a z-score algorithm with a threshold of 2.5. Most experiments were performed in duplicate by swapping dyes between test and control.

### Validation of array results

Copy number aberrations identified by array CGH and SNP array were validated by FISH, M-FISH and M-BAND using protocols described in MacKinnon *et al. *[18]. Metasystems XCyte probes (Metasystems, Altlusshem, Germany) for M-FISH and M-BAND were used according to the manufacturer's instructions.

M-BAND of chromosome 2 was used to confirm the copy number aberrations detected in the cell line U937, using the XCyte 2 probe (Metasystems, Altlussheim, Germany). M-BAND probes are chromosome-specific and produce a multicolor banded chromosome pattern. The XCyte 2 probe is comprised of eight partially overlapping region-specific paints labeled with different fluorochromes. The Isis algorithm converts relative fluorescence intensities into false colors, creating a multicolored banding pattern where each region of chromosome 2 is identified by a unique color. Comparison of the pattern on the abnormal chromosomes with the normal chromosome 2 pattern showed deletion of 2qter and inverted duplication of the adjacent segment in one abnormal chromosome and partial loss of 2p in the other.

Deletions of 20q were confirmed by FISH with a probe for the common deleted region (Vysis LSI D20S108 (20q12) SpectrumOrange, Abbott Molecular, Abbott Park, Ill.). Chromosome 5 deletions were confirmed by M-FISH and/or M-BAND and a Vysis probe detecting 5q31 deletions (LSI EGR1 (5q31) SpectrumOrange/D5S721, D5S23 SpectrumGreen, Abbott Molecular). Deletion of 7q was confirmed by Vysis LSI D7S486 (7q31) SpectrumOrange/CEP7 SpectrumGreen (Abbott Molecular).

This study was approved by Human Research Ethics Committee A of St Vincent's Hospital (Melbourne) Ltd, Protocol HREC-A 091/02, and complies with the Helsinki Declaration.

## Competing interests

The authors declare that they have no competing interests.

## Authors' contributions

RNM carried out DNA extraction, microarray and FISH studies. CS carried out RNA extraction and analysis. AZ and MW assisted with the SNP array studies. CS, AZ, MW, HN and LJC contributed advice and feedback. RNM wrote the manuscript with input from co-authors. All authors read and approved the final manuscript.
